# Mental disorder and caregiver burden in spouses: the Nord-Trøndelag health study

**DOI:** 10.1186/1471-2458-10-516

**Published:** 2010-08-26

**Authors:** Mariann Idstad, Helga Ask, Kristian Tambs

**Affiliations:** 1 Norwegian Institute of Public Health, Division of Mental Health, PO Box 4404 Nydalen, N-0403 Oslo, Norway

## Abstract

**Background:**

Researchers generally agree that mental disorder represents a burden to the family. The present study concerns the subjective burden of living with a person with mental disorder, more specifically the association between mental disorder in the index person and subjective well-being and symptoms of anxiety and depression in the spouse.

**Methods:**

Data were obtained from questionnaires administered to the adult population of Nord-Trøndelag County, Norway during the period 1995-1997. The present study is based on a subsample where 9,740 couples were identified. Subjective burden in spouses of persons with mental disorder was compared with subjective burden in spouses of persons without mental disorder, using analysis of variance (ANOVA). All analyses were stratified by sex.

**Results:**

Adjusting for several covariates, spouses of persons with mental disorder scored significantly lower on subjective well-being and significantly higher on symptoms of anxiety and depression compared to spouses of index persons without mental disorder. Although highly significant, the effect sizes were moderate, corresponding to a difference in standard deviations ranging from .34 - .51.

**Conclusions:**

Our study supports the notion that there is an association between mental disorder in one partner and subjective burden in the spouse, but not to the same extent that have been reported in earlier studies, as our results do not indicate that a large proportion of the spouses reach a symptom level of anxiety and depression that reflects clinical mental disorder.

## Background

The global burden of mental disorder is increasing [1]. Depression alone is reported to be one of the leading causes of disability worldwide, accounting for 4.4% of lost years of healthy life due to premature death or disability (DALYs) on a global basis [2] and 6.2% of all DALYs in the European Region [1]. Prevalences for mood- and anxiety disorders within a 12-month period have been estimated to 9.5% and 18.1%, respectively, in the United States [3]. Similarly, in Norway almost 10% of the adult general population has been suffering from a depression disorder and almost 20% from anxiety disorders during a 12 month period [4]. Prevalence for anxiety and depression in the European Region at any point in time has been estimated to 100 million people, corresponding to 11.5% of the population [1]. The economic costs of affective and anxiety disorders to society are substantial, amounting to €147 billion in the European Region in 2004 [5].

Researchers generally agree that mental disorder represents a burden to the caregiver and family (for reviews, see [6-10]). The interest in caregiver burden emerged when mentally ill patients started to be deinstitutionalized [11]. Caregiver burden refers to the significant amount of strain and difficulties experienced by the caregiver or family of mentally ill people, including a range of psychological, emotional, social, physical and financial problems [6,8,12-14]. In the literature, definitions distinguish between subjective and objective burden [15], and this distinction has established itself as a general guideline for researchers in the field [16]. The former includes perceived psychological distress such as feelings of loss, embarrassment in social situations, and depression, whereas the latter includes the practical and concrete problems such as reduced social and family activities and financial difficulties. The present study focuses on the part of subjective burden that concerns psychological distress, observed as symptoms of anxiety and depression and low subjective well-being.

Research on caregiver burden has traditionally focused on relatives of individuals with severe mental disorders like schizophrenia (for reviews, see [10,16,17]), bipolar disorder (for reviews, see [18,19]), and dementia (for review, see [20]). Although more disorders have been included in recent research [21], there are few studies on families of individuals suffering from anxiety and depression disorders, and it has been pointed out that there is a lack of research based on large sample sizes and control groups [19,22-24].

The individuals classified with mental disorder in the present study are selected on the basis of a high score on symptoms of anxiety and depression, and having sought professional help for mental health problems and/or having reduced functionality as a consequence of mental health problems. Although some of these individuals may be suffering from schizophrenia or bipolar disorder, such cases are likely to be few, as people with severe mental disorder tend to be underrepresented in population based studies. It is likely that the majority of individuals classified with mental disorder are predominately cases of anxiety and/or depression, as these are known to be the mental disorders with the highest prevalence. However, as the case group may be somewhat heterogeneous regarding type of illness, the cases will be referred to as having "mental disorder". The present study investigates caregiver burden in spouses, more specifically the association between mental disorder in the index person and symptoms of anxiety and depression and subjective well-being in his or her spouse. As mentioned above, the spousal symptoms of anxiety and depression and subjective well-being represent subjective caregiver burden in our study, and will thus be referred to as "subjective burden". Earlier studies on this subject reported that living with a depressed person puts the spouse at risk of experiencing elevated levels of psychological distress and depression and represents considerable strain on the marriage [23,25-30].

Studies reporting that the burden of depression is smaller or more infrequent compared to the burden of for example bipolar disorder [31], schizophrenia [23] or dementia [32] may lead to a general perception that burden is larger for the more severe psychiatric diagnoses; however, other studies comparing the burden of depression with the burden of schizophrenia [24] or dementia [33] found similar amounts of burden. Furthermore, a study of partners of people suffering from anxiety disorders, depression or schizophrenia did not find any support for a relationship between strength of burden and type of diagnosis or duration of the illness, but rather between burden and level of impairment in everyday functioning [34]. However, in this study there was a selection bias; the spouses chosen for participation all had partners who were undergoing institutional treatment and thus had severely impaired everyday functioning.

There are mixed findings in the literature regarding the association between caregiver's age and burden; it has been suggested that this might be due to differences in the intensity of the relative's illness in each study so that crisis conditions may produce a greater burden regardless of age, whereas stable conditions may not produce a great burden in elderly caregivers due to more experience in dealing with the illness [6]. According to this notion, we expect elderly partners to report a lower amount of burden than younger partners because the individuals classified with mental disorder in the present study are likely to suffer from anxiety and depression disorders that tend to recur and thus make for a fairly stable condition. In a similar vein, we expect partners who have been married for many years to report a lower amount of burden than do partners who have not been married for many years.

A priori it is far from obvious that there exists a sex difference in the strength of subjective burden. On one hand, it is well established that women tend to suffer from depression to a greater extent than men, which may imply that they are more vulnerable to certain burdens, but on the other hand, women also tend to have larger social networks and receive more social support. Some previous evidence shows that female spouses of mentally ill husbands tend to report greater levels of depression than vice versa (see e.g, [35]), however, one meta-analysis focusing predominately on dementia caregivers found small to very small gender differences [36].

The literature on caregiver burden on affective disorders and spousal mental health overlaps with other literature concerning depression in couples. For example, a recent review on health concordance within couples [37] reported overwhelming evidence for concordant couple mental health, especially regarding depressive symptoms, and showed that affective contagion is one of the explanations that are most frequently used. Assortative mating - that people tend to select life partners that share similar characteristics as themselves - is also a possible explanation of observed spouse similarity for mental health [37]. Furthermore, depression in spouses is strongly associated with marital distress, and new models are now studying the importance of the interpersonal aspects of depression, such as social interaction and marital quality [38]. Unfortunately, our data do not permit us to integrate these perspectives into our study.

Based on previous research, we hypothesize that there is an association between mental disorder in the index person and increased levels of symptoms of anxiety and depression and decreased levels of subjective well-being in the spouse, compared to couples in which the index person does not suffer from mental disorder. Furthermore, we expect elderly partners to report a lower amount of burden than do younger partners, and equivalently, partners who have been married for many years to report a lower amount of burden than do partners who have been married for a few years. We will also explore eventual gender differences. To the best of our knowledge, the present study is the first population based study to investigate the association between anxiety and depression disorders in one spouse and mental health in the other spouse within the field of caregiver burden research.

## Methods

### Sample

The present study is based on data from the Nord-Trøndelag Health Study (HUNT) in Norway. Approval to use the data was provided by the HUNT Research Centre. The population in the county of Nord-Trøndelag is fairly representative of the Norwegian general population. All inhabitants in Nord-Trøndelag county above the age of 19 were invited to participate. The present study is based on HUNT 2, the second wave of data collection, which was carried out in 1995-97. Out of the 94,194 individuals that were invited to the study, a total of 92,936 were eligible for participation, of which 66,140 participated (71.2%). A questionnaire, Q1, was attached to the mailed invitation and returned at the examination site. A second questionnaire, Q2, was handed out during the examination and returned some days after by pre-paid mail. 57,315 of the participants (86.7%) returned Q2. The sample is described in detail elsewhere [39].

In a subsample of HUNT 2, including 17 of the 24 municipalities in the county, data on an abbreviated version of SCL-25 [40] were available. 51,574 persons (62.8%) participated, and the subjects' age ranged from 20 to 101 years (mean = 50.2, SD = 17.0). A detailed description of the sample is available elsewhere [41]. The governmental statistics agency, Statistics Norway, used the 11-digit personal identification number assigned to Norwegian citizens to identify registered couples. For the purpose of the present study, only individuals from couples with valid data on the outcome measures *symptoms of anxiety and depression *(SCL) and *subjective well-being *(SWB) were included, resulting in a sample of 9,740 mixed-sex couples (48.8% of all the couples invited). Characteristics of the sample are reported in the results.

### Measures

#### Symptoms of anxiety and depression

Symptoms of anxiety and depression during the last two weeks were measured by ten of the 25 items in the Symptom Checklist-25 [40]. Four questions tap anxiety (Suddenly scared for no reason; Feeling fearful; Faintness, dizziness, or weakness; Feeling tense or keyed up) and six tap depression (Blaming yourself for things; Difficulty in falling asleep or staying asleep; Feeling blue; Feeling of worthlessness; Feeling everything is an effort; Feeling hopeless about future). Response categories range from "not at all", scored 1, to "extremely", scored 4. This ten item version has been observed to correlate .97 with the original version, in an available subsample from HUNT 1 [42]. Cronbach's alpha was .84 for men and .86 for women.

#### Criteria of mental disorder in the index person

The data in HUNT 2 are not informative on who meets the criteria of a diagnosis. Thus, strict criteria including both SCL and other information were developed in order to identify index persons highly likely to suffer from mental disorder. A dichotomous combined variable (case/not case) was coded as positive if the following two criteria were met: 1) A score of 20 (corresponding to an average score across items of 2.0) or higher on the SCL (range 10-40). This criterion is stricter than the mean score of 1.75 across items commonly used as a SCL-25 cut off score for "severe depression". 2) A score of 1 or higher on an indicator of mental health problems consisting of two items; "reduced functionality due to mental health problems" (range 1-3: "A little", "quite a deal", "a lot") and "having sought professional help for a mental health problem" (range 0-1, no/yes). We added this criterion in order to be reasonably sure that we would correctly identify cases of mental disorder and avoid false positives. The choice of such a strict combination of criteria inevitably causes some loss of true positive cases from the case group to the non-case group. However, this modest contamination of the non-case group cannot substantially change the difference between the two groups. This resulted in a total of 540 cases; 357 women and 183 men, corresponding to 2.8% of the present sample, which is substantially lower than regular prevalence estimates [4]. The low prevalence rate, implying that our criteria are strict, indicates that our case group is relatively free from false positives.

Data from a previous HUNT study taking place 11 years earlier on a crude indicator of symptoms of anxiety and depression described elsewhere [42], were available for 23,095 of the HUNT 2 participants. To further ensure that we had managed to identify cases with severe and relatively persistent mental disorder, we investigated whether the cases also had a high score on anxiety/depression in HUNT 1. Even 11 years earlier the cases on average scored 1.48 SD higher on anxiety/depression compared to the other population.

#### Subjective Well-Being

Subjective well-being was measured by a three-item indicator that has been proven reliable in previous studies (i.e, [43]). Answers were given on a 7-point scale ranging from "very satisfied" to "very unsatisfied". The questions were phrased as follows: *When you think about your life at the moment, would you say that you are by and large satisfied with life, or are you mostly dissatisfied?*; *Would you say you are usually cheerful or dejected?; *and *Do you mostly feel strong and fit, or tired and worn out?*. The items were standardized and added as a sum score indicator. Cronbach's alpha was .77 for both men and women.

#### Control variables and confounders

The association between poor mental health in the index person and the mental health in his or her spouse may be confounded by a number of variables. We adjusted for spouses' age, education, years of marriage, and whether the spouses were also living together with persons under 18 years of age. Covariates also included somatic disease and alcohol problems in both index persons and spouses due to the possible confounding effect on spousal mental health. A sum score indicator was calculated for a number of somatic diseases including infarction, angina, stroke, diabetes, difficulty in breathing, epilepsy, cancer, other long term disease, and impairment of motor ability, vision, hearing or somatic illness. Alcohol consumption was measured with four items: *How many times a month do you usually drink alcohol?*; *How many glasses of beer, wine or liquor do you usually drink in the course of two weeks? *(separate responses for each category). A summative indicator was computed in which frequency and total amount of units were equally weighted.

### Treatment of missing values

We used SPSS Missing Value Analysis (MVA), expectation maximization (EM) for imputation of missing values in respondents with valid data for at least half of the items. For SCL, the ten items were used as predictors for each other. Likewise the SWB items and alcohol items, respectively, were used to predict each other. Missing values (at least one item missing) were reduced from 15.3% to 8.9% for the SCL indicator, from 4.9% to 1.6% for the SWB indicator, and from 12.3% to 2.6% for the alcohol consumption indicator. Regarding years of marriage/cohabitation, valid data were available only for married couples and not for unmarried couples, who corresponded to 8.1% of the present sample. Missing values were reduced to 0.1% by placing respondents younger than 35 years in the category "married less than 10 years", respondents 35-44 years in the category "married 10-20 years" and respondents 45 years and up in the category "married more than 20 years". This classification was empirically tested in pairs with valid data on marital duration and gives 17% misclassification. If the unmarried couples are similar to married couples in terms of duration of the relationship, then 268 individuals would be misclassified according to this classification. 2.7% of the respondents did not report level of education and were placed in the lowest level of education. Missing values for the variable "Living with persons under 18 years" were placed in the category "not living with persons under 18".

### Design and statistical analyses

The present study applies a cross-sectional design, investigating subjective burden, observed as low subjective well-being and symptoms of anxiety and depression, in spouses of index persons suffering from mental disorder. Multivariate ANOVA (SPSS General Linear Models, Unianova) was conducted for each of the two outcome measures, SCL and SWB. Mental disorder in the index person was entered as a dichotomous factor (case or not a case), adjusting for spousal age, education, years of marriage, living together with persons under 18 years of age, and also adjusting for somatic disease and alcohol problems in both index persons and spouses. Because of the double entry file structure (each participant in the study was included both as an index person and as a spouse), all analyses were run stratified by sex. Thus, one set of analyses estimated the association between mental disorder among male index persons and subjective burden in their female spouses, while the other estimated the relation between mental disorder in female index persons and subjective burden in thier male spouses.

An association between mental disorder in the index person and spousal subjective burden might vary with the spouses' own age, years of marriage, and whether they are living together with persons under 18 years of age. Accordingly, interaction terms between mental disorder in the index person and the spousal variables were tested.

The SCL scores were highly skewed, and were ln-transformed to obtain closer to normal distributions. The dependent variables, SCL and SWB, were standardized before used in the analyses. The unstandardized regression coefficients (b) therefore show adjusted group mean differences scaled in fractions of a standard deviation (SD) for the dependent variables.

## Results

Mean age was 53.4 years (SD = 14.42) for men and 50.8 years (SD = 14.26) for women. Among males, 35.9% were in the age group 20-44 years, 45.5% in the group 45-64 years, and 18.6% in the group 65 years or more. The corresponding percentiles for women were 43.5%, 43.7%, and 12.8%. Mean score on SCL (range 10-40) was 11.89 (SD = 2.93) for men and 12.83 (SD = 3.65) for women. Mean score on SWB (range 1-7) was 5.18 (SD = .86) for men and 5.09 (SD = .87) for women.

The correlation between husbands' and wives' logarithmically transformed SCL scores was 0.16, and the correlation between husbands' and wives' SWB score was 0.26.

Analyses of variance were run consecutively with the two outcome variables, spousal SCL and spousal SWB, in both sex strata. Being a mental disorder case was significantly associated with spousal scores on SCL and SWB for both male and female spouses, and this association remained significant after controlling for spousal age, education, years of marriage, living together with persons under 18 years of age, and somatic disease and alcohol problems in both index persons and spouses. The nonadjusted and adjusted differences between the groups in fractions of SDs (b) are presented in Table 1.

**Table 1:** Relation between mental disorders in the index person and spousal symptoms of anxiety and depression (SCL) and subjective well being (SWB)

**Outcome variable and model**^**a**^	N pairs	N cases	**b (CI) **^**b**^	**η**^**2 c**^
Males				
SCL NA	9,733	352	.39 (.29 -.49)	.006
SCL A	9,300	339	.34 (.24 - .44)	.005
SWB NA	9,733	352	-.47 (-.57 - -.36)	.008
SWB A	9,300	339	-.39 (-.49 - -.28)	.006
Females				
SCL NA	9,740	178	.59 (.44 - .75)	.006
SCL A	9,310	174	.51 (.35 - .66)	.004
SWB NA	9,740	178	-.54 (-.69 - -.39)	.005
SWB A	9,310	174	-.42 (-.57 - -.27)	.003

^a ^NA = non adjusted scores, A = adjusted scores

^b ^Unstandardized regression coefficient (b) with 95% confidence interval (CI). The coefficients show adjusted mean deviations from spouses of persons without mental disorder in fractions of a standard deviation, p < 0.001 for all coefficients.

^c ^Partial Eta Squared.

### Interaction effects

Interaction terms were specified between the case's mental disorder and the spouse's age, years of marriage, and living together with persons under 18 years of age, respectively. No significant interaction effects were detected.

## Discussion

The present study aimed at investigating the association between mental disorder in the index person and spousal subjective burden, based on a large, population based sample. Because the study is based on cross-sectional data, we cannot make causal inferences, however the results show a clear association. Female spouses of cases with mental disorder scored about half a SD higher on SCL and male spouses scored about one third of a SD higher on SCL compared to the remaining population, and both male and female spouses scored more than one third of a SD lower on SWB. Although highly significant, these are moderate effect sizes. Testing for interaction effects did not yield any significant results, which may be due to a lack of power, as very large sample sizes are required for these kinds of analyses.

### Mental disorder and subjective caregiver burden

Because the majority of mental disorders in the community are recurrent anxiety and depressive disorders [4], it is possible that our findings are largely attributable to index persons with these disorders. Our results show that the spouses of these persons report higher levels of symptoms of anxiety and depression and lower levels of subjective well-being compared to the other population, and may thus support earlier studies which found that spouses of depressed individuals are at risk of developing depression themselves [23,25-30].

As we did not have the possibility of identifying cases of for example bipolar disorder, schizophrenia or dementia, we could not compare the burden of anxiety and depression disorders with the burden of any of these disorders. This makes it difficult to relate our results to findings from other studies comparing the burden of different disorders. However, it could be argued that the moderate effect sizes found in our study indicate that anxiety and depression disorders are not associated with a very heavy burden, and that this lends some support to previous results showing a smaller burden associated with depressive disorders than other disorders [23,31,32].

Furthermore, the cases in the present study were selected on the basis of strict criteria reflecting recurrent mental health problems, and our results could thus be compared with the study which concluded that it was the degree of impairment and not diagnosis per se that caused burden in the spouses [34]. Even though we have probably succeeded in identifying cases with severely impaired daily functioning, the effect sizes were moderate.

In sum, the present study shows that there is an association between mental disorder in one person and spousal symptoms of anxiety and depression. This supports the notion that mental disorder may represent a burden to the spouse, but not to such a great extent that has been indicated in some earlier studies (e.g, [34]). The moderate effect sizes in the present study do not at all imply that a large part of the spouses of the cases reach a symptom level of anxiety and depression that reflects clinical mental disorder. What is more, as opposed to clinical studies, population based studies may yield more valid results because the participants are unaware of the study's purpose and thus, are not inclined to produce responses biased by being reminded and focused on a negative aspect of their life. However, the results show a clear association which should not be ignored. Imperfect measurement precision and sample selection against depressed persons may have deflated the estimates. Furthermore, even if mental disorder *on average *did not appear to be strongly related to spousal subjective burden, it may well be in some couples.

### Gender differences

It is well known in the literature that women tend to suffer from anxiety and depression more frequently than men. This is also true for the present study; the female cases with mental disorder outnumbered their male counterparts. But, interestingly, the association between mental disorder in the index person and spousal subjective burden appears to be similar in men and women. Although the difference in SCL score between wives of husbands with or without mental disorder was larger than the difference in SCL score between husbands of wives with or without mental disorder, the moderate effect sizes in sum show a similar pattern. This supports earlier findings of small to very small gender differences [36] and indicates that although more women than men tend to suffer from anxiety and depression, the strength of the association between mental disorder in the index person and spousal subjective burden is similar in men and women. It does make sense that living with a depressed person may be associated with subjective burden in the partner regardless of his or her gender.

### Methodological considerations

As mentioned earlier, population based studies concerning anxiety and depression disorders in one partner and caregiver burden in the spouse are practically absent in the literature to date. The present study is based on a large, representative sample and the dependent variables are based on well known, validated measures. Mental health was measured both by SCL and SWB; these measures may be claimed to essentially represent opposite ends of the same positive/negative affectivity dimension. Still, applying both may yield more complete information because SCL is mainly sensitive to the pathological part of the affectivity distribution, whereas the SWB scores are sensitive also to the happy part of the distribution. Consequently, our study yields important information considering mental disorder and subjective burden in spouses. However, there are some limitations. The data are not based on diagnoses; hence it is not possible to safely establish whether all index persons classified as cases actually do suffer from a clinical mental disorder. Nevertheless, strict criteria used in identifying individuals with mental disorder probably imply that the vast majority of the persons classified as cases have severe mental health problems. It is also impossible to determine the proportion of cases who suffer from even more serious illnesses than anxiety or depression, like bipolar disorder or schizophrenia, but it is not likely that more than a small fraction of individuals with such diseases have participated in the survey. Although the results show a significant association between mental disorder in the index person and spousal subjective burden, it is not possible to conclude about the causal direction due to the study's cross-sectional design. Another limitation is the lack of validated instruments designed to measure the extent of caregiver burden; however, psychological distress is clearly included in the definition of subjective burden. Likewise, we were not able to separate couples who receive help and support from couples who do not receive any help, thus the association in the couples without support may be stronger than shown by our results.

## Conclusions

The present study shows that spouses of persons who are suffering from mental disorder report significantly higher levels of symptoms of anxiety and depression and significantly lower SWB than do spouses of persons not suffering from mental disorders, although the effect sizes are moderate. This supports earlier research regarding caregiver burden and depression in spouses. However, our results do not indicate that a large proportion of the spouses reach a symptom level of anxiety and depression that approaches clinical mental disorder. Moreover, the results in the present study indicate that the strength of the association between mental disorder in the index person and spousal subjective burden is similar in male and female spouses. Although issues of caregiver burden are receiving increased attention, there is still a strong need of studies of the burden of mental disorders, particularly anxiety and depression. Researchers studying mental disorder and caregiver burden in couples should also, contrary to what our own data permit, try to address alternative explanations of partner resemblance in mental health, like assortative mating, effects of marital quality, and circularity of symptomatology in which symptoms in the spouse as a consequence of symptoms in the index person in turn may worsen the index person's symptoms. Disentangling the different sources of spouse resemblance would contribute to a better and more comprehensive understanding of the dynamics and causes of caregiver burden. More epidemiological and longitudinal studies and studies from different cultures are needed.

## Competing interests

The authors declare that they have no competing interests.

## Authors' contributions

MI performed the statistical analyses and drafted the manuscript. All authors contributed to the study's design, preparation of the data, interpretation of analyses and helped to draft or critically revise the manuscript. All authors read and approved the final manuscript.

## Pre-publication history

The pre-publication history for this paper can be accessed here:

http://www.biomedcentral.com/1471-2458/10/516/prepub
